# The relationship between trait empathy and memory formation for social vs. non-social information

**DOI:** 10.1186/s40359-015-0058-3

**Published:** 2015-02-03

**Authors:** Ullrich Wagner, Lisa Handke, Henrik Walter

**Affiliations:** Department of Psychology, University of Münster, Fliednerstr. 21, 48149 Münster, Germany; Division of Mind and Brain Research, Department of Psychiatry and Psychotherapy, Charité – Universitätsmedizin Berlin, Campus Mitte, Charitéplatz 1, 10117 Berlin, Germany

**Keywords:** Cognitive empathy, Affective empathy, Social memory, Perspective taking, Personal distress

## Abstract

**Background:**

To navigate successfully through their complex social environment, humans need both empathic and mnemonic skills. Little is known on how these two types of psychological abilities relate to each other in humans. Although initial clinical findings suggest a positive association, systematic investigations in healthy subject samples have not yet been performed. Differentiating cognitive and affective aspects of empathy, we assumed that cognitive empathy would be positively associated with general memory performance, while affective empathy, due to enhanced other-related emotional reactions, would be related to a relative memory advantage for information of social as compared to non-social relevance.

**Methods:**

We investigated in young healthy participants the relationship between dispositional cognitive and affective empathy, as measured by Davis’ Interpersonal Reactivity Index (Journal of Personality and Social Psychology, 44, 113–126, 1983), and memory formation for stimuli (numbers presented in a lottery choice task) that could be encoded in either a social (other-related) or a non-social (self-related) way within the task.

**Results:**

Cognitive empathy, specifically perspective taking, correlated with overall memory performance (regardless of encoding condition), while affective empathy, specifically empathic personal distress, predicted differential memory for socially vs. non-socially encoded information.

**Conclusion:**

Both cognitive and affective empathy are associated with memory formation, but in different ways, depending on the social nature of the memory content. These results open new and so far widely neglected avenues of psychological research on the relationship between social and cognitive skills.

## Background

Humans are highly social animals. Successful navigation through our social life within our cultural environment is a demanding task, requiring constant tracking and interpretation of others’ behavior. A central psychological skill that humans have developed to meet these demands is empathy, the ability to understand and to share the mental states of others, which encompasses cognitive empathy (mentally representing other’s thoughts, feelings, and intentions, frequently also called perspective taking, mentalizing, or “Theory of Mind”) and affective empathy (aligning one’s own with another person’s emotional state) (Davis, 1983; Baron-Cohen and Wheelwright, 2004). Another critical skill that we constantly need in our social life relates to memory. In order to interact appropriately within society, we must not only be able to remember other individuals per se, but also how they have behaved in the past, and how we have behaved towards them in previous interactions. Are our mnemonic abilities associated with empathy? And if so, does this relationship apply to both cognitive and affective empathy, and is it specifically conducive to social memory, i.e. stronger in social as compared to non-social encoding contexts? Evolutionary accounts proposing a tight interaction between of cognitive and social factors in the extraordinary development of the human brain within primate evolution (e.g., Dunbar and Shultz 2007; Pinker 2010) would indeed suggest such associations.

Still, relatively little is known about answers to these questions, probably because in basic psychological research, memory and empathy have traditionally been treated as research topics belonging to different domains of psychology, i.e. cognitive psychology and social and personality psychology, respectively. However, there are initial clinical findings that indeed point to a positive relationship between empathy and memory functions. Specifically, patients primarily characterized by memory deficits appear to concomitantly show reduced empathic abilities, in particular in cognitive empathy, as has been reported in dementia patients (Cuerva et al. 2001; Gregory et al. 2002; Zaitchik et al., 2006; Fernandez-Duque et al. 2010). In these studies, patients with Alzheimer’s disease (and in the study by Fernandez-Duque et al. additionally also patients with frontotemporal dementia) were assessed in tests in which they had to explicitly infer the beliefs, feelings or thoughts of another person from film material of an interview with the other person or from verbal descriptions of false belief stories. In false belief stories, originally developed by Perner and Wimmer (1985) in the context of developmental psychology, participants have to recognize that a target person has a different view on or state of knowledge of a scene than oneself or other persons. These tests of “Theory of Mind” can be varied in difficulty, depending on the level of perspective-taking that they require. Alzheimer patients as well as patients with frontotemporal dementia were impaired in these perspective-taking tasks, especially when these were difficult. Additional findings suggested that the deficits were related to other cognitive impairments in these patients, including their memory impairments, which were most pronounced in those subjects who did not pass the difficult perspective-taking tests (Cuerva et al., 2001; Zaitchik et al., 2006). Although little focus has been on the aspect of emotional empathy in these clinical studies, at least one study also reported deficits in this domain in Alzheimer patients (Laisney et al., 2013), using the Reading the Mind in the Eyes Test by Baron-Cohen et al. (2001), which requires participants to recognize the emotional state of a person form a picture that only shows the eye region of that person.

Finally, a study by Beadle et al. (2013) assessed empathy in another type of patients with memory impairment, i.e. amnesic patients with hippocampal damage. These authors did not apply formal behavioral tests like the false belief task, but assessed dispositional empathy of the patients, as measured by the Interpersonal Reactivity Index (IRI) from Davis (1983). This trait questionnaire, comprising four subscales, likewise distinguishes between cognitive and affective aspects of empathy, with two subscales (perspective taking and fantasy) representing the former one and the two other subscales (empathic concern and personal distress) representing the latter one. The amnesic patients showed reduced empathy on all scales, which was most pronounced for cognitive empathy (perspective taking). In parallel to the reduced trait empathy, the same patients also showed attenuated responses to experimental empathy induction in comparison to healthy controls (Beadle et al., 2013). These deficits may be the reason for inappropriate character judgments and reduced social networks observed in such amnesic patients (Croft et al., 2010; Davidson et al., 2012).

Here, we aimed to extend these initial findings from the clinical domain by investigating the relationship between specific aspects of empathy and memory in a sample of healthy participants. Although the clinical results provide valuable information on how different psychological mechanisms are related to each other, we think that it is important to extend this field also into basic research in healthy populations to get a clearer picture on the normal processes that determine the relationship between processes of empathy and memory formation. This is desirable for several reasons. First, the basic mechanisms in patients may differ qualitatively from those in healthy subjects, either due to the disturbances in the neural system or due to compensatory mechanisms. Thus the concomitant lack in two domains in neurologically disturbed patients does not necessarily imply that the processes in these two domains are also connected to each other under normal circumstances. Second, most patient studies are inherently investigating only participants of older age, and especially in studies based on case reports of patients, some abnormalities could also result of idiosyncratic circumstances in certain individuals. Third, although several studies as cited above suggest a relationship between empathy and memory, at least one patient study has been performed that suggests independence of these two domains (Rosenbaum et al., 2007), so it is worthwhile to specify the relationship directly in a sample of young healthy participants.

Hence, we present here a study investigating the relationship between dispositional empathy, as measured by the IRI mentioned above, and the encoding of new information in memory in a sample of healthy students. As an additional novel aspect, not yet considered in previous studies, we also took, apart from memory performance per se, the social factor of memory encoding into account, i.e. whether information is encoded in a social (other-related) or in a non-social (self-related) way. Memory was tested in a standard recognition test for the socially vs. non-socially encoded information. We expected the social factor to play an additional important role, because highly empathic participants should be more sensitive towards social information than participants with low empathy. With regard to memory supporting mechanisms, such increased sensitivity towards social information should be effective to the extent in which it induces emotional processing of stimuli, because an emotional way of stimulus encoding is well-known to promote memory formation (Christianson 1992). We therefore expected specifically affective empathy, which is defined by emotional empathic reactivity towards others, to be positively associated with a relative memory advantage for social as compared to non-social memory encoding, although we did not anticipate which of the two facets of affective empathy assessed by the IRI (i.e. empathic concern or personal distress) would primarily show this association. However, regarding memory performance per se (regardless of social or non-social encoding conditions), we expected a specific association with cognitive empathy, and in particular perspective taking, which would confirm the general picture from the clinical studies cited above. Finally, we also introduced the factor of active vs. passive encoding, which has been reported to affect memory encoding (e.g., Watanabe and Soraci, 2004) and might therefore also be a moderator for the relationship between dispositional empathy and memory as assessed here.

## Methods

### Participants

Twenty-three healthy participants (all white, 10 female) without a history of neurological or psychological disorders were recruited at the Charité Universitätsmedizin Berlin and the Free University Berlin for the experiment. Participants’ ages ranged between 19 and 31 years (mean = 23.61 years). Participants gave informed written consent and were paid for participation. The study was approved by the ethics committee of the Department of Psychiatry and Psychotherapy at the Charité Universitätsmedizin Berlin, Campus Mitte.

### Procedure

**Figure 1:**
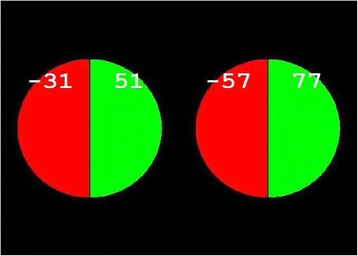
**Example of a lottery choice slide presented during memory encoding.** Each circle represents a lottery, which could have a financial gain (positive numbers on green part of a circle) or a financial loss (negative numbers on red part of a circle) as an outcome. A choice had to be made which of the two lotteries would actually be played. In half of the trials, the financial gain or loss of the lottery played was assigned to the participant (non-social encoding condition), in the other half to another person in need (social encoding condition; see text, for details). In an active choice condition, participants selected the lottery to be played themselves, while in a passive choice condition, the computer made the choice randomly. (A slide in the beginning of each trial announced the respective experimental condition of the trial, i.e. whether it was social vs. nonsocial and whether it was active or passive). After the choice had been made, the chosen lottery was marked, and finally the outcome of both lotteries was shown. Memory for all numbers presented in the lottery task was assessed in a subsequent recognition memory test. During performance of the lottery choice task, participants were not aware that a recognition memory test for the numbers would follow.

Participants first performed a decision-making task as described in detail previously (Wagner et al. 2012), in which numbers – indicating potential gains or losses of lotteries – were processed in either a social or a nonsocial experimental condition. In each trial, a choice had to be made between two “wheel of fortune” lotteries (Figure 1), each featuring a win and a loss outcome with corresponding outcome probabilities. The probabilities of the possible financial gain or loss were represented by the relative size of colored sectors of a circle (green for win probabilities and red for loss probabilities). The extent of the possible gains and losses in a given trial was indicated by positive numbers on the green part of the respective circle (for the possible gains) and negative numbers on the red part of the respective circle (for the possible losses). These numbers represented Euro cents that could be won or lost in the respective lottery, which could be up to 500 cents per trial, i.e. presented values ranged between −500 and 500.

Critically, in half of the trials this amount of money was assigned to the participant (self-related = non-social condition), while in the other half of the trials it was assigned to another person (other-related = social condition). For all participants, the specified beneficiary of all the earnings in the social condition was a 4-year old Ukrainian girl in need of medical treatment, for which a local charity organization was collecting donations. Participants received detailed information about the organization and about the Ukrainian girl and her disease in the beginning of the experiment. For this purpose, they were shown the website of the organization presenting the project, and they also read a printed one-page summary. The money won for the Ukrainian girl in the experiment was actually transferred to the account of the charity organization afterwards. To ensure that participants believed this, they were informed in advance that in the end of the experiment, they would sign not only the money transfer form for themselves, but also for the donation to the charity organization collecting money for the child.

In addition to this factor of social vs. non-social processing, half of the trials were presented in an active condition (with participants choosing the lottery themselves), the other half in a passive condition (with participants observing the computer randomly choosing between lotteries). Altogether, the experiment comprised 112 trials (i.e. 28 trials in each of the four conditions social/active, social/passive, non-social/active, non-social/passive), presented in random order. In each trial, participants first read the corresponding experimental condition on a text slide for 2.5 sec before the two possible lotteries were shown. After one of the two lotteries had been chosen, this lottery was marked on the screen, and subsequently the outcome of both the chosen and the non-chosen lottery were shown. Importantly, the task was constructed in such a way that each specific gain or loss number was presented in only one task trial. Thus, participants processed altogether 448 different numbers (four per trial) ranging between −500 and +500. Amounts divisible by 50 were avoided because such numbers could “pop out” and therefore be inherently more memorable than other numbers. Furthermore, to counterbalance any number-specific memory effects, assignment of specific lottery pairs to experimental conditions was balanced across participants.

The decision-making task served as the phase of (incidental) memory encoding of the numbers displayed in the lotteries. The critical memory test followed immediately thereafter as a surprise recognition memory test. In this memory test, all the 448 numbers shown in the previous decision-making task were successively presented again, intermixed with additional 360 new numbers (likewise ranging between −500 and +500) and in random order. For each of the numbers participants had to indicate whether it was old (presented in one of the trials during the decision-making task) or new (not presented in the decision-making task).

To assess individual dispositional empathy, participants filled in the Interpersonal Reactivity Index (IRI) from Davis (1983) in a shortened German version provided by Paulus (2009). As mentioned, the questionnaire contains four different empathy scales, with two covering cognitive aspects of empathy (Perspective Taking = adopting the mental perspective of another person, and Fantasy = adopting the perspective of a fictional character in a book or film), and two covering emotional aspects of empathy (Empathic Concern = experiencing feelings of compassion and sympathy for others, and Personal Distress = feeling unease or distress in the face of physical or emotional harm of another person). Example items are (in English): “Before criticizing somebody, I try to imagine how I would feel if I were in their place” (Perspective Taking); “When I watch a good movie, I can easily put myself in the place of the leading character (Fantasy); “When I see someone taken advantage of, I feel kind of protective toward them” (Empathic Concern); and “I sometimes feel helpless when I am in the middle of a very emotional situation” (Personal Distress). Responses are given for each item on a 5-point scale ranging from 0 to 4. In the German version that we used each scale is represented by 4 items, so that the maximum score for each of the four scales is 16.

## Results

### Overall memory performance and empathy

**Figure 2:**
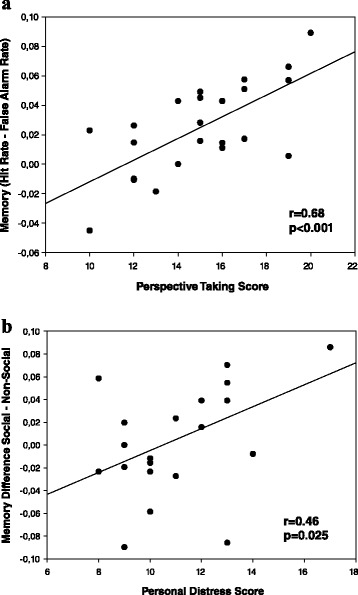
**Relationship between empathy and memory performance (difference hit rate – false alarm rate). (a)** Perspective Taking correlated with overall memory performance (p < 0.001), **(b)** while Personal Distress correlated with differential memory for socially vs. non-socially encoded numbers (difference social memory – non-social memory, p = 0.025).

Memory performance was calculated as the difference between hit rate (percentage of previously shown numbers correctly identified as “old”; 44.3% ± 2.8%) and false alarm rate (percentage of new numbers incorrectly identified as “old”; 41.8% ± 2.6%), representing a response-bias independent memory index (Snodgrass and Corwin 1988). This index was overall clearly higher than zero (2.48% ± 0.64%, t(22) = 3.85, p < 0.001, d = 0.80), confirming substantial residual memory for the numbers, despite the relatively high task difficulty. Overall memory performance (across encoding conditions) correlated substantially with Perspective Taking (r = 0.68, p < 0.001; Figure 2a), but not with the other empathy scales of the IRI (Fantasy, r = 0.18; Empathic Concern, r = 0.21; Personal Distress, r = −0.12; all p > 0.33).

### Social vs. non-social memory and empathy

Our second analysis referred specifically to the *difference* in social vs. non-social memory in relation to empathy. Accordingly, we calculated for each participant the difference between memory for socially and non-socially encoded numbers as the critical dependent variable. (A positive value of this variable indicates an advantage of social over non-social memory, while a negative value indicates an advantage of non-social over social memory encoding.) This indicator of social memory advantage showed a significant positive correlation with Personal Distress (r = 0.46, p = 0.025; Figure 2b), but not with the other IRI scales (Perspective Taking, r = −0.19; Fantasy, r = 0.05; Empathic Concern, r = −0.06, all p > 0.39). Interestingly, the regression line as depicted in Figure 2b also crosses the zero line of the x-axis, showing that the overall pattern reverses as Personal Distress increases. That is, while subjects with low Personal Distress show an overall memory advantage of self-related over other-related information, subjects with high Personal Distress show an overall memory advantage of other-related over self-related information. Accordingly, in the group as a whole, there was no overall advantage of social over non-social memory (0.49% ± 0.99%, t(22) = 0.50, p = 0.63, for difference from zero).

We additionally performed the following secondary analyses:

*Active vs. passive choosing condition.* We explored in an additional 2×2 ANOVA the possibility of an interaction of the effects of Personal Distress with whether the choice of the lottery was actively performed or passively observed (factor “Active/Passive”), dichotomizing the empathy factor Personal Distress by median split into high and low scorers. (Two participants scoring exactly on the median of Personal Distress were excluded from this analysis for optimal discrimination between groups.) This ANOVA confirmed a significant main effect of Personal Distress (F(1,19 = 6.11, p = 0.023, η_p_^2^ = 0.24, reflecting the positive correlation mentioned above), but Personal Distress did not interact with the factor “Active/Passive” (F(1,19) < 1). Thus, the effect of Personal Distress on memory did not depend on whether the information on possible outcomes was encoded under active or passive choice conditions. Independent of Personal Distress, the memory difference between social and non-social memory tended to be lower for numbers from active trials than from passive trials (F(1,19) = 2.98, p = 0.10, η_p_^2^ = 0.13, for main effect “Active/Passive”).

*Lottery outcome.* Inherent to the lottery game, part of the numbers, i.e. those finally drawn as lottery outcomes, were shown again in the end of the trials, increasing both their psychological relevance and their overall encoding time in comparison to other numbers. Therefore, in another complementing ANOVA, we further explored the possibility that the effect of Personal Distress depended on whether numbers were actually realized as lottery outcomes or not. For this purpose, the factor Outcome (outcome numbers vs. non-outcome numbers) was included as an additional within-subjects factor. There was an overall main effect of Outcome, with enhanced social memory superiority for realized compared to non-realized numbers (F(1,19) = 7.10, p = 0.015, η_p_^2^ = 0.27). However, Personal Distress did not interact with this factor, neither alone (F(1,19) = 0.10, p = 0.76, for Personal Distress x Outcome interaction) or in combination with the factor “Active/Passive” (F(1,19) = 0.07, p = 0.80, for Personal Distress x Outcome x Active/Passive interaction).

*Response times.* We also performed an ANOVA on response times, i.e. the time participants needed to choose their preferred lottery, as an additional control analysis to rule out that the results could be explained simply by differences in encoding times between conditions. (The two lotteries were shown on the screen as long as participants needed to decide – although with an upper limit of 8 seconds, but this was rarely exceeded –, so longer response times would also result in longer opportunity to encode the respective numbers). This control analysis confirmed that response times (overall averaging 3.49 sec) did not depend on Personal Distress, Self/Other, or Active/Passive conditions (p > 0.12, for all main effects and interactions). Thus, the memory effects could not simply be attributed to differences in encoding times between conditions.

*Gender.* Women typically show higher empathy than men (Davis, 1983; Baron-Cohen and Wheelwright, 2004). Thus, our results could theoretically simply reflect gender difference in empathy, rather than genuine effects of empathy. In the present study, women indeed scored higher than men in Perspective Taking (12.4 ± 0.73 vs. 9.92 ± 0.79, t(21) = 2.24, p = 0.036, d = 0.98; similarly for Fantasy, 11.4 ± 0.67 vs. 8.08 ± 0.94, p = 0.013, d = 1.14, and Empathic Concern, 11.7 ± 0.73 vs. 9.38 ± 0.74, p = 0.040, d = 0.92), but there was no difference between women and men in Personal Distress (7.40 ± 0.88 vs. 6.69 ± 0.51, t(21) = 0.73, p = 0.47). Thus, a gender confound would be possible here for the result of a correlation between Perspective Taking and overall memory performance. However, when we calculated this correlation separately for both sexes, the relationship remained strongly positive within each of the two subgroups (r = 0.68, p = 0.031, for women; r = 0.76, p = 0.003, for men). Furthermore, an ANOVA on memory performance including gender as a between-subjects factor together with the within-subjects factors Self/Other and Active/Passive revealed no significant effect involving gender (all p > 0.18).

## Discussion

We investigated the relationship between empathy, as measured by Davis’ (1983) IRI, and social vs. non-social memory formation in healthy humans. The social factor in memory formation was defined as the way in which otherwise comparable stimuli (here numbers) were encoded, namely as either relevant to oneself (non-social condition) or to another person (social condition). Two main results were obtained, indicating differential involvement of cognitive and affective aspects of empathy in memory formation. First, Perspective Taking, a prototypical measure of cognitive empathic capabilities, correlated with overall memory performance regardless of encoding conditions. Second, and specific to the social vs. non-social nature of encoding, Personal Distress, a measure of affective empathic reactions, correlated with the relative advantage of social as compared to non-social memory encoding. Control analyses showed that these findings could not be accounted for simply by other factors affecting encoding, such as encoding time or active vs. passive encoding.

Our result of a positive relationship between Perspective Taking and general memory performance is in line with clinical findings reporting reduced empathy, specifically in the cognitive domain, in neurological patients with primary memory impairments (Cuerva et al. 2001; Gregory et al. 2002; Beadle et al. 2013; Fernandez-Duque et al. 2010). Here, we confirm and extend these results by demonstrating a positive correlation between Perspective Taking and general memory performance in a non-clinical sample of young healthy participants. This finding is also in line with the only previous study we are aware of that directly addressed in a sample of healthy participants the relationship between a behavioral measure of perspective taking and the extent of memory encoding of experimentally presented material (Stiller and Dunbar, 2007). Although these authors were primarily interested in the independent predictive values of perspective taking and memory performance for participants’ social network sizes, their data also show that the two cognitive abilities were strongly associated with each other, consistent with our findings here based on dispositional perspective taking, with likewise large effect sizes.

Importantly, our data additionally point to a specific role of affective empathy and particularly Personal Distress with regard to the social factor in memory encoding. That is, not Perspective Taking, but Personal Distress appears to be associated with sensitivity specifically to the social nature of information in the context of mnemonic processing. In fact, participants high in Personal Distress tended to remember socially encoded information better than non-socially encoded information, while participants with low Personal Distress showed a result in the opposite direction, consistent with the typical “self-reference effect” in memory, i.e. better memory when participants encode items in a way forming associations in relation to themselves than in another semantic manner (Symons and Johnson 1997).

Our finding that affective but not cognitive empathy was associated with *differential* encoding of social vs. non-social information is in line with our idea that emotional factors determine these differential effects. Specifically, although our experiment was not designed to reveal the underlying mechanisms by which empathic capabilities affect memory encoding, we assume emotional arousal as a critical underlying factor. Participants with high affective empathy, more than those with low affective empathy, are likely to react with enhanced emotional arousal specifically in the social as compared to the non-social gamble situation. Emotional arousal is well known to enhance memory formation, an effect that is neurobiologically linked to amygdala activation and related neurophysiological mechanisms (e.g., LaBar and Cabeza 2006; Wagner and Born 2008). This explanation fits with the fact that the relative benefit of social memory was specifically associated with the affective empathy aspect of Personal Distress, defined by feelings of personal anxiety and unease in tense interpersonal settings, but not Empathic Concern, defined by feelings of sympathy and concern towards unfortunate others, which represents a calmer and hence presumably less physiologically arousing type of affective reactivity (Davis 1983). Thus, in particular participants with high dispositional empathic Personal Distress may have been more emotionally aroused, with concomitant amygdala activation, when they knew that a gamble affected the fate of the other person rather than themselves, leading to enhanced encoding for the numbers presented. Hence, our data suggest that mechanism by which empathy-related processes can specifically enhance the encoding of social memory contents are basically relying on the same mechanism as memory-enhancing effects of emotion in general, i.e. emotional arousal. While this idea needs to be tested in future neuroscientifc studies more directly, there is an interesting parallel to conclusions about overlapping mechanisms in empathy and memory domains from studies relating to retrieval of memory (as opposed to memory encoding on which we focus here). Markedly overlapping brain networks in tasks of autobiographical memory retrieval and tasks of perspective taking (as well as tasks of future imagination) in frontal, medial temporal and posterior parietal areas have led to the idea that these processes rely on a common mechanism of self-projection, where the self has to be decontextualized from the current situation (Buckner and Carroll, 2007; Spreng and Mar, 2012). Interestingly, a recent study also showed that when participants read about a person in need, imagining a vivid scenario of helping the person as well as remembering a related past event from their own life in which they had helped, led to subsequent increase in their actual willingness to help (Gaesser and Schacter, 2014).

Our data suggest that such overlap may be analogously relevant at the encoding stage, and draw specific attention to the emotional aspects of empathy-related processing with regard to the encoding of socially relevant information. Because empathic concern is frequently regarded as the most prototypical aspect of affective empathy, one could ask why personal distress but not empathic concern was most strongly associated with the superior encoding of social vs. non-social information. However, assuming arousal induced by personal distress as an underlying mechanism, our data are in line with conceptual discussions in which personal distress is regarded as “empathic overarousal”, associated with enhanced physiological reactions which entail stronger self-focus rather than concern for the other (Eisenberg and Fabes, 1990; Eisenberg, 2000). On this basis, these and other authors have distinguished between empathy (basically an equivalent of the term “empathic concern” as used here, by some authors also referred to as “sympathy”) on the one hand, and personal distress on the other hand (Batson et al. 1983; Eisenberg, 2000; Carrera et al., 2013). The issue in how far personal distress conceptually belongs to or is distinct from empathy cannot be resolved within this paper, but we think that it is at least appropriate to refer to personal distress, if based on the suffering of others, as an empathy-related process.

### Limitations

A major limitation of our study is the small sample size, which makes it necessary to confirm our findings in future replications. However, in the relatively novel research field of empathy-memory relationships, it provides incremental information in a sample of healthy adults, for which available data are currently sparse. By focusing on memory encoding, and in this context distinguishing between encoding of social and non-social information, the study also introduces new aspects not yet considered in previous accounts of the topic. A specific methodological advantage of our study design is that it allowed us to vary the social vs. non-social nature of stimuli to be encoded strictly by experimental manipulation. That is, the exactly same stimulus (with no inherent social meaning) could be encoded in either a social or a non-social way. Thereby, confounds by different features of the stimuli themselves could be ruled out. However, it is also to be noted that our task differed from more usual memory paradigms also by other specificities. For example, each trial was associated with real monetary consequences (for either the self or another person). Also, we intentionally presented a child in need of medical treatment as the “other person”, rather than someone anonymous, in order to create a particularly empathy-conducive situation in the social condition. It is conceivable that effects of the large size that we were able to detect here even with a relatively small sample size, and consequently low power, would not necessarily occur with more subtle manipulations of the social factor in memory. Therefore, the generalizability of our present findings should be tested with different memory tasks and larger samples sizes.

Another limitation is that our assessment of empathy was confined to self-report measures as provided by the IRI. However, clinical studies demonstrate that patient groups showing disturbed empathy as measured by the IRI also perform worse in objective tests of empathy and social behavior as well as in judgments of patients’ empathic skills by relatives (Cusi et al. 2010, 2012; Shany-Ur et al., 2012; Beadle et al., 2013), and direct correlations of IRI measures with objective empathy measures in healthy subjects confirm a generally positive relationship (Rogers et al. 2007; Dziobek et al. 2008). These findings indicate that biases to which self reports may be more susceptible than other measures (e.g., social desirability) do not challenge their general validity. Still, future studies further examining the relationship between empathy and memory should also include objective tests of empathy. Another aspect to be further explored in relation to memory processing would be state empathy, i.e. transient empathic reactions to specific situations, rather than dispositional trait empathy that was in the research focus here. Furthermore, especially from the perspective of basic research that we are adopting here, more non-clinical studies would be needed to circumvent the interpretation problems that arise from data obtained in patient groups, as mentioned above (see also Choong and Doody 2013).

## Conclusion

Together, the present study provides the first systematic investigation of the relationship between empathic and mnemonic abilities in healthy humans, distinguishing between social and non-social memory formation. We show that cognitive empathy (specifically Perspective Taking) is associated with higher memory performance in general (in line with previous conclusions derived mostly from clinical studies), while affective empathy (specifically Personal Distress) is associated with enhanced differential memory performance for social in comparison to non-social information. The underlying mechanisms are still to be specified, as well as the generalizability of the results. On a broader view, this research strengthens new scientific directions that attempt to reveal how social factors influence memory processes (Echterhoff and Hirst, 2009).
